# An Analysis of Disparities in Access to Health Care in Iran: Evidence from Lorestan Province

**DOI:** 10.5539/gjhs.v6n5p81

**Published:** 2014-05-13

**Authors:** Reza Nemati, Hesam Seyedin, Ali Nemati, Jamil Sadeghifar, Ali Beigi Nasiri, Seyyed Meysam Mousavi, Keyvan Rahmani, Mostafa Beigi Nasiri

**Affiliations:** 1 Department of Geography, Faculty of Humanities, Tarbiat Modares University, Iran; 2 Health Management and Economics Research Center, School of Health Management and Information Sciences, Iran University of Medical Sciences, Tehran, Iran; 3 Hospital Management Research Center, Iran University of Medical Sciences, Tehran, Iran; 4 Research Center for Health Services Management, Institute for Futures Studies in Health, Kerman University of Medical Sciences, Kerman, Iran; 5 Department of Health Education, School of Public Health, Ilam University of Medical Sciences, Ilam, Iran; 6 Jawaharlal Nehru Technological University, Hyderabad, India; 7 Research Center for Modeling in Health, Institute for Futures Studies in Health, Kerman University of Medical Sciences, Kerman, Iran; 8 Department of Health Management and Economics, School of Public Health, Tehran University of Medical Sciences, Tehran, Iran; 9 Shahid Chamran University, Ahvaz, Iran

**Keywords:** disparity, healthcare, access, scalogram analysis model, Iran

## Abstract

Equal distribution of healthcare facilities in order to increase the accessibility of the individuals to services is one of the main pillars in improvement of health. This study was aimed to examine the disparities in access to health care services across the cities of Lorestan province located in west of Iran. This study is a descriptive study. Data related to indicators of institutional and manpower was collected using statistical yearbook of Statistical Centre of Iran (SCI) and analyzed by Scaogram Analysis Model. The results revealed distinct regional disparities in health care services across Lorestan province. According to Scalogram analysis model, Khorramabad and Delfan towns were ranked as the first and the last according to access to health care services. Overally, 44% of the cities are undeveloped and only 22% are credited as developed. Taking the advantage of development-oriented programs, reduction of the gap in health care services in the must be considered in the health policy. Therefore, Delfan, Dorood, Koohdasht and Selseleh are characterized as the underdeveloped and consequently urgently should be considered in planning and deprivation programs.

## 1. Introduction

To achieve human and economic sustainable development, it is necessary to minimize disparities (Zarrabi & Shaykh Baygloo, 2011). Various studies have been conducted with different attitudes on regional disparities. These studies have been mostly conducted using economic and social indices and infrastructure development indices to classify different geographical areas (Poor Fathi & Asheri, 2010). GDP and GDP per capita are the main indicators to assess development level. These indicators do not consider fairness in the distribution of health services and other social services (Mahmoudi, 2011). Health and development are closely linked to each other and affect interchangeably (Rafi’iyaan & Taajdaar, 2008).

Health sector plays a decisive role in the wellbeing of people (A. Sayemiri & K. Sayemiri, 2001). Access to health care is crucial and is a multi-dimensional concept; physical access and financial access. Physical access is defined as geographical access to health facilities which can affect health services usage (Paez, Mercado, Farber, Morency, & Roorda, 2010) andis concern of community and health policy makers (Amaral, 2009; Comber, Brunsdon, & Radburn, 2011). Regional studies in different countries show that specific areas enjoy the modern facilities and have better performance (Amini, Yadolahi, & Inanlu, 2006).

After the Islamic Revolution in Iran special attention is paid to the health sector. Iran’s Constitution, has defied the provision of basic needs in health care as the responsibility of the government to mobilize its resources to meet the nation’s health (Ahmadi, Ghaffari, & Emadi, 2011). The geographical distribution of health indicators (as one of the most important indicators of development) in the cities of Iran is heterogeneous and disproportionate (Taghvaei & Shahivandi, 2011). Iran’s geographical conditions, has led to the diversity and unbalanced development (Ghanbari, 2011). Similar to other developing countries, some areas compare to small areas are responsible for the majority of production and national income. This means their income is in higher level and as a result they enjoy more public service (Mohammadi, Abdoli, & Fathi Biranvand, 2012). Therefore, it is necessary to define conditions of access to health services and then develop a comprehensive program to fix the problem.

**Figure 1:**
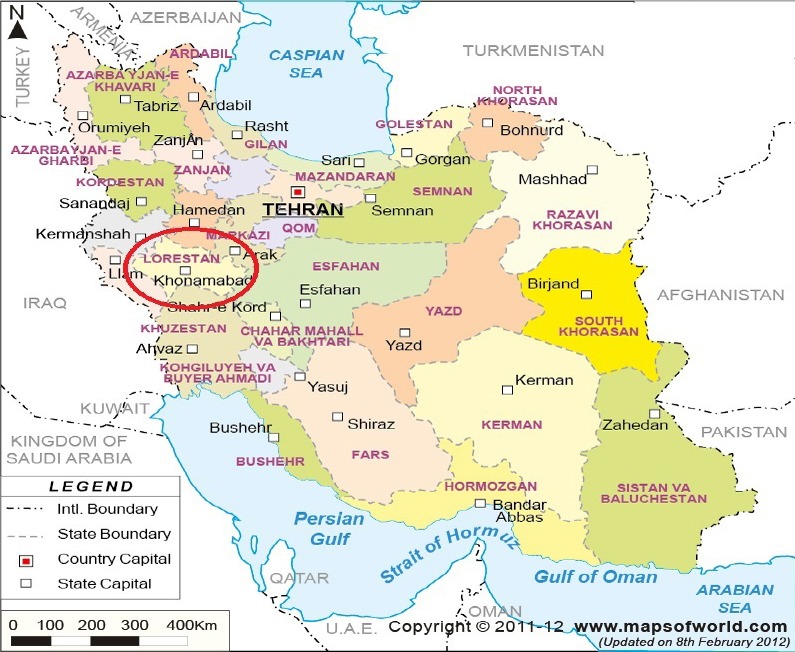
Political map of Iran

Iran, a mountainous, arid, ethnically diverse country of western Asia (Afary, Avery, & Mostofi, 2013). It is surrounded on the west by Iraq; on the northwest by Turkey; on the east by Afghanistan and Pakistan; on the north by Armenia, Azerbaijan and Turkmenistan, with Kazakhstan and Russia through the Caspian Sea; and on the south by the Persian Gulf and the Gulf of Oman (Figure 1).

Lorestan Province is a province in west of Iran. It borders Markazi and Hamedan provinces to the north, Kermanshah and Ilam provinces to the west, Khuzestan province to the south and Isfahan province to the east. This study was conducted using Scalogram analysis model to assess the regional disparities in terms of health services access across the Lorestan province.

## 2. Materials and Methods

In this descriptive analytical study, Lorestan’s cities are graded with respect to their accessibility of healthcare facilities using the Scalogram analysis model. The data gathered in two groups of institutional and manpower indices using a data-gathering questionnaire, from statistical yearbook and data related to population of cities as gained from archives of Statistical Centre of Iran (SCI). These indices were selected due to availability of statistical date for them. The data analysis was conducted based on Scalogram analysis model.

The Scalogram analysis model is employed in a scientific process including index selection, calculation of mean, and finally distinguishing the gap between classes and town gradation (Bahadori, Shams, Sadeghifar, Hamouzadeh, & Nejati, 2012; Mousavi et al., 2013):

Phase 1: In the first phase, 32 healthcare structural indices were selected using the data from the annual demography of the province. The availability, structural nature and comments of three experts in the Health Policy were taken as factors. The selected indices were classified to two groups of institutional and manpower indices according to the experts’ opinion (Table 1).

**Table 1:** Structural indices extracted from the statistical yearbook of Lorestan province

Institutional indices	Manpower indices
Number of active treatment centers per 1000 people (A), Number of active beds of treatment centers per 1000 people (B), Number of health centers per 1000 people (C), The ratio of public health centers to active health center (D), The ratio of day-working health centers to the active health centers (E), The ratio of round health centers to active health center (F), Number of laboratory per 1000 people (G), Number of pharmacy to 1000 people (H), Number of radiology centers per 1000 people (I), Number of rehabilitation centers to 1000 people (J)	Number of internist per 1000 people (K), Number of cardiologist per 1000 people (L), Number of pediatricians per 1000 people (M), Number of psychiatrists per 1000 people (N), Number of dermatologist per 1000 people (O), Number of general surgery per 1000 people (P), Number of urologist per 1000 people (Q), Number of orthopedist per 1000 people (R), Number of neurologist per 1000 people (S), Number of ENT specialist per 1000 people (T), Number of eye specialist per 1000 people (U), Number of anesthesiologist per 1000 people (V), Number of pathology per 1000 people (W), Number of dentist per 1000 people (X), Number of pharmacologist per 1000 people (Y), Number of infection specialists per 1000 people (Z), Number of general practitioner per 1000 people (AB), Number of paramedical per 1000 people (AC), Number of dental health worker per 1000 people (AD), Number of nurses (BSc or higher education) per 1000 people (AE), Number of anesthesiology technicians (BSc or lower education) per 1000 people (AF), Number of mental therapists per 1000 people (AG)

Phase 2. In the second phase, the mean (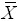) and Standard Deviation (SD) of the indices were calculated at towns’ level.

Phase 3: In the third phase, the upper and lower limit of each and every index was determined based on the formulas (
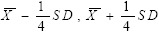
). If the number is above the upper limit, the condition is perceived as positive and is graded as 3. If the number is less than the lower limit, the condition is negative and is considered equal 1. In case the index number falls between the upper and lower limit, the condition is neutral and it is graded as 2. The positive status exemplifies the developed index, the negative one indicates the deterioration index and the neutral position symbolizes the normal index in the specific town. Therefore, regarding the 32 indices, the maximum and minimum grades of enjoyment of the overall indices equals 96 and 32 for each town, respectively.

Phase 4: In the fourth phase, to determine the developing gap in the healthcare structural indices among the towns, 5 levels of developed, some developed, moderately development, less developed and underdeveloped were described; Following the calculation of the overall index grades in a town, the variability range (R) of the grades were calculated and the interval between classes were measured using Sturges’ formula:


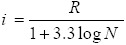


*i* = class interval

R= class range

N= number of the cases to be classified

Following the calculation of the class intervals, Lorestan’s towns were classified to five classes according to their overall grades of access of healthcare structural indices.

## 3. Results

First, the raw data of indices in Lorestan province for determining the degree of development of cities is derived (Table 2).

**Table 2:** The raw data of indices in Lorestan province

	Institutional indices										Manpower indices																						T-Scores
**Indices**	A	B	C	D	E	F	G	H	I	G	K	L	M	N	O	P	Q	R	S	T	U	V	W	X	Y	Z	AB	AC	AD	AE	AF	AG	

**Aligoodarz**	1	3	3	3	2	3	2	1	3	3	3	1	2	1	1	3	3	1	3	3	3	1	1	3	2	3	2	2	1	2	3	1	69
**Borujerd**	1	2	1	3	3	1	1	3	2	3	1	3	1	1	3	2	1	3	2	2	3	2	3	3	3	2	2	1	1	3	1	1	64
**Khorramabad**	3	3	1	1	3	1	2	3	3	1	1	3	1	3	3	2	3	3	3	2	3	1	2	2	3	3	3	3	2	3	1	2	73
**Dekfan**	1	1	1	1	3	1	1	1	1	1	1	3	2	1	1	1	3	1	1	3	1	1	3	1	1	1	1	1	3	1	3	2	48
**Dorood**	2	2	1	2	1	1	1	3	3	2	1	3	2	1	1	2	1	3	1	3	1	1	1	1	1	1	1	1	1	2	1	1	49
**Koohdasht**	1	1	1	3	1	3	1	1	1	1	1	2	1	3	1	1	1	3	3	1	3	1	1	1	2	1	1	1	3	1	2	1	49
**Azna**	3	1	3	3	2	2	3	3	2	3	3	1	2	1	1	3	1	1	1	1	1	3	1	3	2	1	3	3	1	1	3	2	64
**Pol-e-Dokhtar**	2	2	3	1	1	3	3	1	2	2	3	1	3	1	1	3	3	1	1	1	1	3	1	3	1	1	3	3	3	3	2	3	65
**Selseleh**	3	1	3	1	2	2	3	1	1	3	3	1	2	1	1	1	1	1	1	1	1	1	1	2	3	1	1	2	3	1	1	3	53

As Table 1 shows, among the 9 cities of Lorestan province, Khorramabad towns with score of 73 and Delfan with score of 48 had the highest (1^th^) and the lowest (9^th^) ranks respectively. Two towns of Khorramabad and Aligoodarz were introduced as developed. Three towns of Pol-e-Dokhtar, Borujerd and Azna were graded as 65 and 64 respectively; with constitute 33.33% of all the townsas relatively developed.

In the present study, undeveloped towns e.g. Selseleh, Koohdasht, Dorood and Delfan gained 53, 49, 49 and 48 respectively, totally making up 44.45% of the towns. The findings reveal that the towns of this group do not enjoy equal distribution of the healthcare facilities (Table 3).

**Table 3:** Status of development in cities of Lorestan province according to health structural indices

Group	Classes distance	Degree of enjoyment	City name	Number	Percent
1	68.01-73	Developed	Khorramabad, Aligoodarz	2	22.22
2	63.01-68	Some deal developed	Borujerd, Azna, Pol-e-Dokhtar	3	33.33
3	58.01-63	moderately developed	-	0	0
4	53.01-58	less developed	-	0	0
5	48-53	Under developed	Delfan, Dorood, Koohdasht, Selseleh	4	44.45

## 4. Discussion

Accessibility of health services is one of the most important indicators for developing countries (Zarrabi & Shaykh Baygloo, 2011). The first step in health development and reducing health gap is complete understanding of the accessibility situation of the regions to health services and facilities. For this reason, the study measured the ranking of Lorestan province cities in terms of having health indicators. The aim of this study was to classify Lorestan province towns in the distribution of health indices using Scalogram analysis model.

This study that was the first to consider health indices in Lorestan province, 44.45 percent of the cities (4 cities) to be classified in the less developed and underdeveloped level and 55.55 percent of them to be classified in developed level. From these cities, Khorramabad and Delfan obtained the highest and lowest enjoyment of health structural indices.

In national (Abolhallaje et al., 2014; Anjomshoa et al., 2014; Mousavi et al., 2013; Sadeghifar et al., 2014; Taghvaei & Shahivandi, 2011) and international studies (Asanin & Wilson, 2008; Boutayeb & Helmert, 2011; Fang et al., 2010; Horev, Pesis-Katz, & Mukamel, 2004; Kreng & Yang, 2011; Peters et al., 2008; Pong, Desmeules, & Lagace, 2009; Theodorakis, Mantzavinis, Rrumbullaku, Lionis, & Trell, 2006) similar results have been obtained in terms of of health indicators.

Generally, the gap in terms of access to health care indices has been observed both among regions of a country or cities of a province. The existence of a balanced development in geographical area and definite development aspects (such as social, political, cultural, economical development) is a necessity.

Policymakers should focus their efforts on finding the reason of development gaps and distinctions and overcoming the allied problems. In order to reduce the existing health inequality between cities and the equal distribution of health care services, it is crucial to develop a national comprehensive plan for moving from macro–scale and top-down program to micro-scale and local plan.

Besides simplicity of the application, the power of the study is that applied tools would obviously show the existing differences in terms of access to resources in local or regional level and therefore the facilities are allocated in a fair approach. What should be important for health policymakers is that the regional disparity in health indices between provinces should be extremely lower than rate of difference of these indices between the cities of a province. Although this topic has not been consider in the research, it leads the policymakers to have more monitoring on allocation of facilities in local levels.

## 5. Conclusion

In order to decrease gap of access to health care between the citiesand distribution of services equitable, concentration on development of health indicators in poor cities (such as Delfan, Doroud, Kouhdasht and Selsele), is recommended. Furthermore, it is suggested that in the first stages of city development, authorities need to focus on short term policies and programs which could result in expansion of critical health services and equity in access and authorities may pay attention to the development of necessary services in developing and deprived cities over a medium and long term plan (5-10 years).
